# P04.72. Towards a model for integrative medicine in the primary care of patients with chronic joint diseases and allergy

**DOI:** 10.1186/1472-6882-12-S1-P342

**Published:** 2012-06-12

**Authors:** M Jong, M Busch, L van de Vijver, E Baars

**Affiliations:** 1 Louis Bolk Institute, Nutrition & Health, Driebergen, Netherlands; 2 van Praag Institute, Utrecht, Netherlands

## Purpose

In the Netherlands, Complementary and Alternative Medicine (CAM) is offered outside the world of mainstream medicine. Patients would benefit from an integrated model in which CAM is part of main stream medicine so that disclosure of risks, benefits of CAM and credentials of CAM practitioners are sufficiently dealt with. The aim of this 4-year research project is to develop, implement and evaluate a shared-care model of Integrative Medicine in a primary care setting for patients with chronic joint diseases and allergy.

## Methods

This is the first project in the Netherlands where patient organizations, health care providers and health care insurers collaborate to achieve an integrative health-care model. The project consists of four stages; in stage 1 needs and preferences of patients were investigated. Based on this outcome, evidence in literature and clinical experiences, an integrated primary care model will be developed (stage 2). The integrated model will be piloted in two primary care centers in the Netherlands (stage 3) and evaluated with respect to outcome (stage 4).

## Results

Stage 1: A national survey demonstrated CAM use in 41% of children with allergy (eczema, asthma) and 71% of adults with chronic joint diseases (arthrosis, rheumatoid arthritis). CAM therapies mostly used were homeopathy, manual therapies, acupuncture and naturopathy. The majority (74% allergy, 51% joint diseases group) did not actively communicate CAM use with their family physician. However, 79% (allergy) and 70% (joint diseases) of patients preferred a physician that informs, refers to and collaborates with CAM.

## Conclusion

CAM use among chronically ill patients is high. Although most patients do not communicate CAM with their family physician, they have a high preference for a shared-care model in primary care. Based on these outcomes, such a model will be developed and presented at the conference.

